# Supplementary material for: Numbers of close contacts of individuals infected with SARS-CoV-2 and their association with government intervention strategies
Source: BMC Public Health. 2021 Dec 9;21:2238. doi: 10.1186/s12889-021-12318-y (PMC8655330; doi:10.1186/s12889-021-12318-y)
Supplement: Supplementary file 1 — Additional file 1. [file 12889_2021_12318_MOESM1_ESM.docx]

**TITLE PAGE**

**Manuscript title**

Numbers of close contacts of individuals infected with SARS-CoV-2 and their association with government intervention strategies.

**Author list**

Conor G. McAloon^1*^, Patrick Wall^2^, Francis Butler^3^, Mary Codd^2^, Eamonn Gormley^1^, Cathal Walsh^4^, Jim Duggan^5^, T. Brendan Murphy^6^, Philip Nolan^7^, Breda Smyth^8^, Katie O’Brien^9^, Conor Teljeur^10^, Martin J. Green^11^, Luke O’Grady^1,11^, Kieran Culhane^12^, Claire Buckley^13^, Ciara Carroll^13^, Sarah Doyle^13^, Jennifer Martin^13^, Simon J. More^1,14^

**Author affiliations**

^1^School of Veterinary Medicine, University College Dublin, Belfield, Dublin 4, Ireland

^2^School of Public Health, Physiotherapy and Sports Science, University College Dublin, Belfield, Dublin 4, Ireland

^3^School of Biosystems and Food Engineering, University College Dublin, Belfield, Dublin 4, Ireland

^4^Department of Mathematics and Statistics, University of Limerick, Ireland

^5^School of Computer Science, National University of Ireland Galway, Galway, Ireland

^6^School of Mathematics and Statistics, University College Dublin, Belfield, Dublin 4, Ireland

^7^National University of Ireland Maynooth, Kildare, Ireland

^8^Department of Public Health, Health Service Executive West, Galway

^9^Department of Health, Dublin 2, Ireland

^10^Health Information and Quality Authority, George’s Court, Dublin 7, Ireland

^11^School of Veterinary Medicine and Science, University of Nottingham, Nottingham, UK

^12^Central Statistics Office, Ardee road, Rathmines, Dublin, Ireland

^13^COVID-19 Contact Management Programme, Health Service Executive, Ireland

^14^Centre for Veterinary Epidemiology and Risk Analysis, School of Veterinary Medicine, University College Dublin, Belfield, Dublin, Ireland

***Corresponding author:**

Conor McAloon,

[conor.mcaloon@ucd.ie](mailto:conor.mcaloon@ucd.ie),

01 716 6083

**Figure S1.** Description of case data entered into the contact tracing database


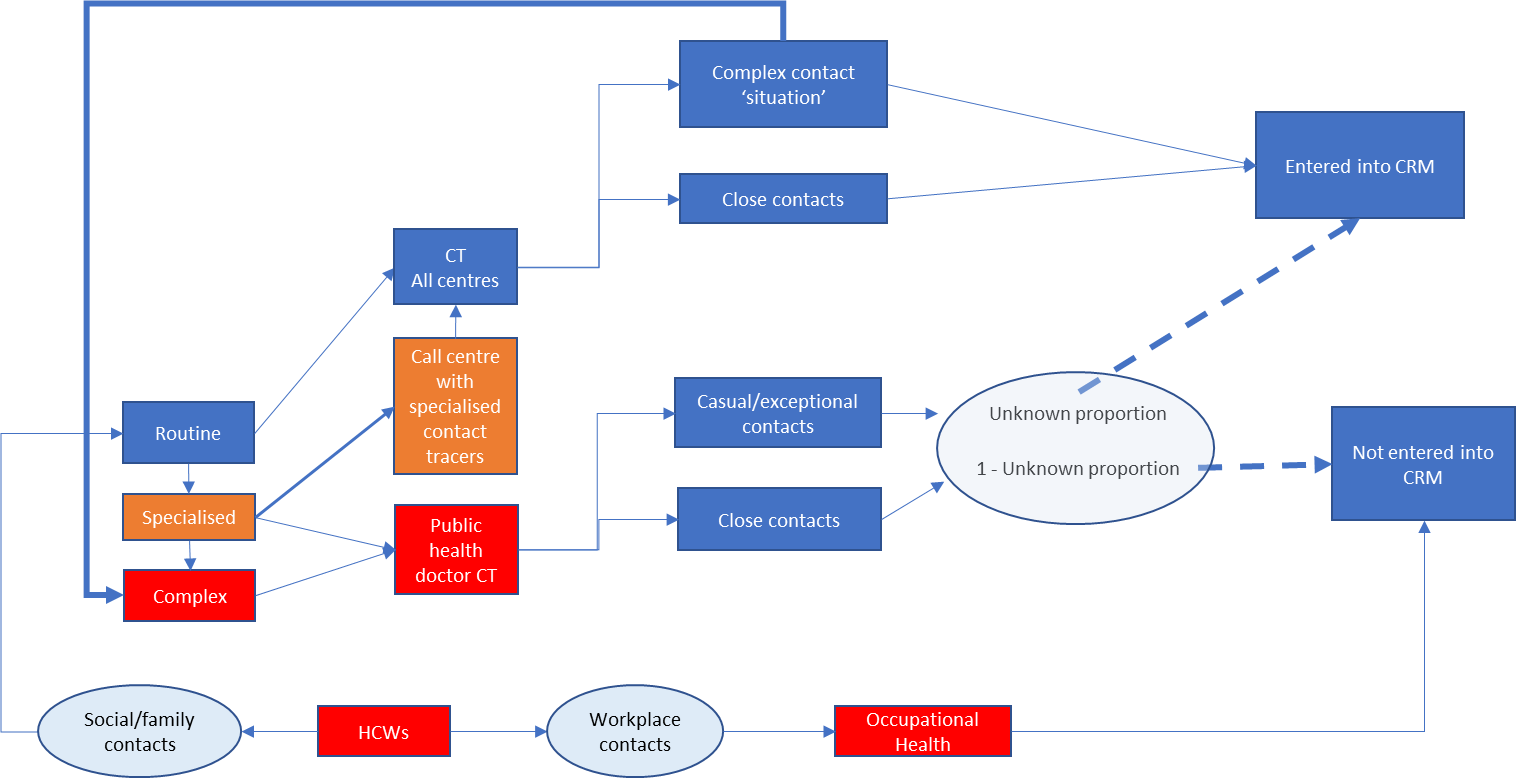


**Figure S2a** Histogram of number of contacts per case prior to filtering to those with less than 50 contacts.

**
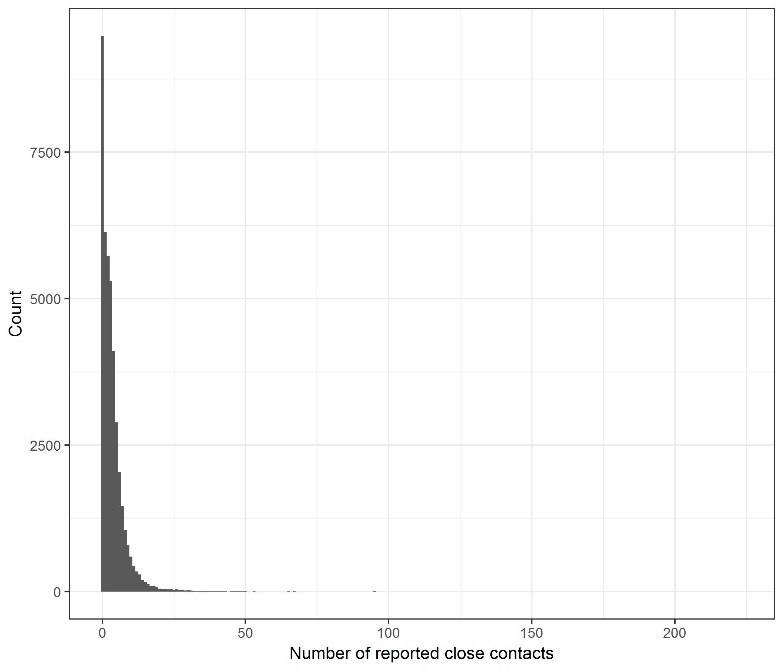
**

**Figure S2b** Histogram of number of contacts per case after filtering to those with less than 50 contacts.

**
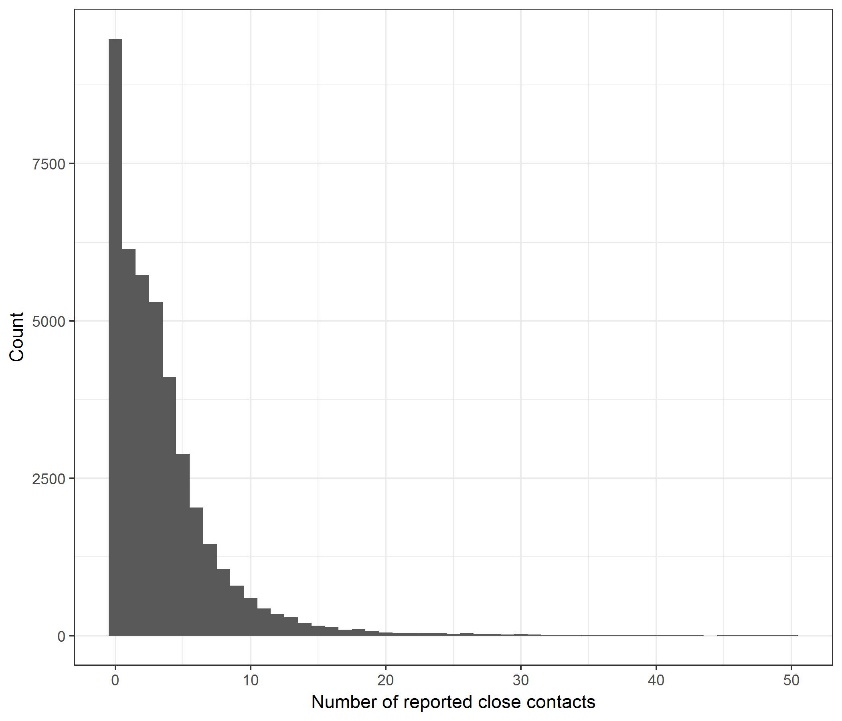
**

**Table S1** Changes in number of records at each stage of data cleaning

| **Data cleaning step** | **Call 1 data (case level)** | **Call 2 data (contact level)** | **Joined data (case level)** | **Joined data (case and contact age cohort level)** |
| --- | --- | --- | --- | --- |
|  |  |  |  |  |
| Initial read in | 79212 | 223651 |  |  |
| Contact Tracing Completed | 60227 |  |  |  |
| Remove duplicate entries | 59181 |  |  |  |
| Drop App recorded contacts |  | 213437 |  |  |
| Drop missing case ID |  | 210090 |  |  |
|  |  |  |  |  |
| Join1 |  |  | 210090 |  |
| Remove contacts with no case |  |  | 205334 |  |
| Remove cases with no or erroneous (age < 0) DOB |  |  | 205300 |  |
|  |  |  |  |  |
| Collapse to case level |  |  | 62472 |  |
| Filter to close contacts |  |  | 42545 |  |
| Join zero contact cases |  |  | 55179 |  |
| Filter within start and end dates |  |  | 41931 |  |
| Filter to contacts less than 50 |  |  | 41900 |  |
| Remove those that do not match to location data |  |  | 39861 |  |
|  |  |  |  |  |
| Collapse to case and age cohort of contact level |  |  |  | 114194 |
| Filter to close contacts |  |  |  | 93746 |
| Reshapeto contact level |  |  |  | 42539 |
| Add zero contact cases |  |  |  | 55173 |
| Convert to long format |  |  |  | 331038 |
| Remove no case age |  |  |  | 330978 |
| Filter within start and end date |  |  |  | 251574 |
| Filter to contacts less than 50 |  |  |  | 251565 |
